# Identification of *Mycobacterium tuberculosis* Infection in Infants and Children With Partial Discrimination Between Active Disease and Asymptomatic Infection

**DOI:** 10.3389/fped.2019.00311

**Published:** 2019-07-25

**Authors:** Alexandra Dreesman, Violette Dirix, Kaat Smits, Véronique Corbière, Anne Van Praet, Sara Debulpaep, Iris De Schutter, Mariet-Karlijn Felderhof, Anne Malfroot, Mahavir Singh, Camille Locht, Françoise Mouchet, Françoise Mascart

**Affiliations:** ^1^Laboratory of Vaccinology and Mucosal Immunity, Université Libre de Bruxelles, Brussels, Belgium; ^2^Pediatric Department, CHU Saint-Pierre, Brussels, Belgium; ^3^Department of Pediatric Pulmonology, Cystic Fibrosis Clinic and Pediatric Infectious Diseases, Universitair Ziekenhuis Brussel, Brussels, Belgium; ^4^Lionex Diagnostics and Therapeutics, Braunschweig, Germany; ^5^INSERM, U1019, Lille, France; ^6^CNRS, UMR8204, Lille, France; ^7^Université de Lille, Lille, France; ^8^Centre d'Infection et d'Immunité de Lille, Institut Pasteur de Lille, Lille, France; ^9^Immunobiology Clinic, Hôpital Erasme, U.L.B., Brussels, Belgium

**Keywords:** *Mycobacterium tuberculosis*, children, diagnosis, latent infection, active tuberculosis, lymphoblasts, FASCIA, dendritic cells

## Abstract

**Background:** Improved diagnostic tests are needed for the early identification of *Mycobacterium tuberculosis-*infected young children exposed to an active TB (aTB) index case. We aimed to compare the diagnostic accuracy of new blood-based tests to that of the tuberculin skin test (TST) for the identification of all infected children and for a potential differentiation between aTB and latent TB infection (LTBI).

**Methods:** 144 children exposed to a patient with aTB were included, and those who met all inclusion criteria (130/144) were classified in three groups based on results from classical investigations: non-infected (NI: *n* = 69, 53%, median age 10 months), LTBI (*n* = 28, 22%, median age 96 months), aTB disease (*n* = 33, 25%, median age 24 months). The first whole blood assay consisted of a 7-days *in vitro* stimulation of blood with four different mycobacterial antigens (40 μl/condition), followed by flow cytometric measurement of the proportions of blast cells appearing among lymphocytes as a result of their specific activation. Thresholds of positivity were determined by Receiver Operating Characteristic (ROC) curve analysis (results of NI children vs. children with LTBI/aTB) in order to identify infected children in a first stage. Other cut-offs were determined to discriminate subgroups of infected children in a second step (results from children with aTB/LTBI). Analysis of blood monocytes and dendritic cell subsets was performed on 100 μl of blood for 25 of these children as a second test in a pilot study.

**Results:** Combining the results of the blast-induced CD3^+^ T lymphocytes by Heparin-Binding Haemagglutinin and by Culture Filtrate Protein-10 identified all but one infected children (sensitivity 98.2% and specificity 86.9%, compared to 93.4 and 100% for the TST). Further identification among infected children of those with aTB was best achieved by the results of blast-induced CD8^+^ T lymphocytes by purified protein derivative (sensitivity for localized aTB: 61.9%, specificity 96.3%), whereas high proportions of blood type 2 myeloid dendritic cells (mDC) were a hallmark of LTBI.

**Conclusions:** New blood-based tests requiring a very small volume allow the accurate identification of *M. tuberculosis*-infected young children among exposed children and are promising to guide the clinical classification of children with aTB or LTBI.

## Introduction

Pediatric tuberculosis (TB) causes significant morbidity and mortality with an estimated 1 million children who became ill with TB and developed active TB disease (aTB) in 2017, 52% of them being <5 years old (1). 230,000 of these children with aTB died from it in 2017, 80% of them were younger than 5 years old (1). These figures are probably an underestimation of the real situation, as there is increasing evidence that many childhood TB cases are not reported or are misdiagnosed (1, 2).

Pediatric TB, like adult TB, is classically subdivided in two different clinical manifestations, latent TB infection (LTBI) and aTB. LTBI is defined as a *Mycobacterium tuberculosis* infection without clinical or radiological signs of disease, whereas in aTB the child presents clinical signs and/or radiological abnormalities. However, in contrast to adults who, in 95% of the cases, develop LTBI after primary infection with *M. tuberculosis*, children, especially those <5 years old, have a higher risk than adults to rapidly progress to aTB, as several known factors necessary to control *M. tuberculosis* infection in adults are immature in young children (3, 4). This implies that most cases of aTB in young children result from progression of a primary infection after a contact with a case of aTB. In contrast, in low TB incidence countries like Belgium where this study was performed, most cases of aTB in adults born in this country result from reactivation of LTBI, who, in the absence of immunosuppression conditions, have an ~5% lifelong risk to reactivate the infection, this risk rising with any medical co-morbidity (3, 5).

Clinical manifestations of aTB, especially in infancy, are often non-specific, mimicking many common childhood diseases, so that diagnosis is often delayed. In addition, microbiological diagnosis of aTB is difficult to obtain, as the disease in children is usually pauci-bacillary. Moreover, even if pulmonary aTB remains the commonest type of aTB in children, young children, especially those younger than 2 years, are at risk to rapidly progress to severe forms of disseminated aTB such as aTB meningitis, associated with high morbidity and mortality (3).

Early identification of *M. tuberculosis-*infected infants and young children is therefore very important and requires systematic detection among children exposed to a case of aTB, most often an adult/adolescent with aTB, as recommended by the World Health Organization (WHO) (1, 6). This implies that each child exposed to an index case of aTB should be evaluated for *M. tuberculosis* infection and classified as infected or non-infected, the demonstration of *M. tuberculosis* infection being a relevant part of the diagnosis of both LTBI and aTB (7). All infected children should then receive appropriate antibiotic treatment, but the decision to provide only a preventive treatment to reduce the risk of developing aTB and becoming infectious, or to treat an established active infection should be based on an accurate differential diagnosis between *M. tuberculosis* infection without disease (LTBI) and aTB.

The tuberculin skin test (TST) remains one of the cornerstones to detect *M. tuberculosis* infection. However, it has important limitations, mostly a lower specificity in case of prior bacillus Calmette-Guérin (BCG) vaccination, in case of infection with non-tuberculous mycobacteria, and possibly in case of repeated TST, referred to as boosting effect (8). TST is also typically unresponsive during the first weeks after infection, especially in infants and in cases of very severe or miliary aTB (9). It does not differentiate children with aTB from infected children who remain healthy and develop LTBI (8). Commercial blood-based immunological tests have more recently been developed to diagnose *M. tuberculosis* infection. They measure the concentration of interferon-γ released (QuantiFERON test) or the numbers of IFN-γ-secreting cells (T-SPOT.TB test) after a short *in vitro* stimulation of blood or peripheral blood mononuclear cells with peptides from two different mycobacterial antigens, the early-secreted antigenic target-6 (ESAT-6), and the culture filtrate protein-10 (CFP-10). These interferon-γ-release assays (IGRA) initially designed to detect LTBI rather than aTB are more specific than TST, especially in BCG-vaccinated subjects, but they also cannot distinguish aTB from LTBI (10), and their sensitivity to detect LTBI is lower than initially thought (11). They clearly cannot be used to reliably rule out LTBI, neither in adults nor in children (8, 11). IGRAs are also increasingly being used as a diagnostic aid for aTB, but even the new QuantiFERON-TB Gold Plus does not provide a sensitivity of 100% for the diagnosis of aTB in adults. This was illustrated in a recent multicenter study, where the overall sensitivity was estimated to be 93%, so that these tests cannot be used to rule out aTB (12). Furthermore, their utility for children, especially for infants, is questionable, as 4 mL blood is needed and as no clear evidence exists for an added value of IGRA over TST for the diagnosis of either LTBI or aTB in children (13, 14).

Other immunological approaches have been evaluated to identify *M. tuberculosis-*infected children and to potentially differentiate those with aTB from those with LTBI. Some of these assays are especially promising for their potential use in infancy, as they are based on the induction of IL-2 secretion by mycobacterial antigens. In contrast to IFN-γ, which production by neonatal CD4^+^ T lymphocytes may be variably impaired, IL-2 is synthesized in appreciable amounts by neonatal lymphocytes (15, 16). One of these approaches is based on the measurement by ELISPOT of the numbers of IL-2-secreting lymphocytes in response to the mycobacterial antigen Rv2780 (secreted L-alanine dehydrogenase), that were significantly higher in children with aTB compared to those with LTBI (17). A potential diagnostic value of IL-2 secretion induced by mycobacterial antigens was also reported by Tebruegge et al. (18), but in this case in response to ESAT-6, CFP-10 or purified protein derivative (PPD), and the results did not discriminate aTB from LTBI. Another IL-2-dependent method was reported earlier to measure lymphoproliferation in response to antigens at a single-cell level (19). This flow cytometric assay for specific cell-mediated immune responses in activated whole blood (FASCIA) was adapted for the diagnosis of TB in adults by measuring the response to ESAT-6 and CFP-10. It provided 86% sensitivity and 91% specificity for the diagnosis of aTB among adults suspected to present aTB (20).

In this study, we adapted this FASCIA method in response to different mycobacterial antigens, i.e., ESAT-6 and CFP-10, associated with the presence of metabolically active bacteria (21), the heparin-binding hemagglutinin (HBHA) inducing stronger immune responses during latency (22) and the mixture of antigens in the form of PPD. The primary aim of our study was to compare the diagnostic accuracy of these tests to the TST for the demonstration of *M. tuberculosis* infection among a cohort of children exposed to an aTB index case as this is a relevant part of the diagnosis of children with either aTB disease or with LTBI (7).The second aim was to evaluate whether, in contrast to the TST, these tests could help the clinician to differentiate, at least partially, children with LTBI from those with aTB among *M. tuberculosis-*infected children. Finally, the third aim was to evaluate in a pilot study, the potential added value for this differentiation between LTBI and aTB of the blood monocyte and dendritic cell (DC) subsets characterization by flow cytometry, based on our recent publication in adults (23).

## Methods

### Ethics Statement

The protocol for this study received approval from the ethics committee ULB-Hôpital Erasme and informed written consent was obtained from all parents.

### Study Population

**Figure 1:**
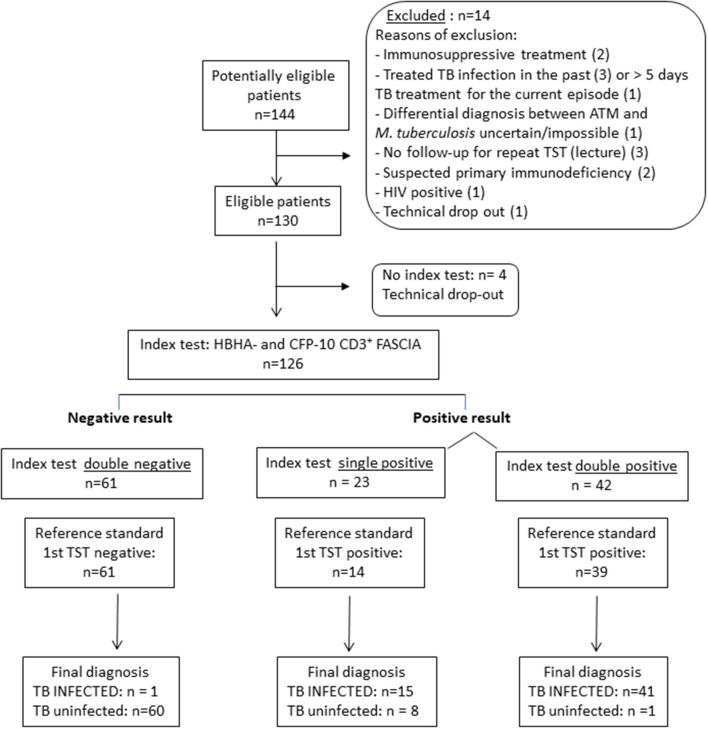
STARD diagram reporting the flow of children for the evaluation of the combined HBHA- and CFP-10-induced CD3^+^ blasts lymphocytes for the identification of *M. tuberculosis-*infected children (LTBI/aTB). The aim of this index test was to correctly classify children exposed to an index case of aTB disease in two groups: non-infected children, and infected children comprising both children with LTBI and those with aTB disease. The reference standard test was the tuberculin skin test (TST) performed at the inclusion of the child in the study (first TST), the TST being the reference test recommended in Belgium and in most European countries at least for children younger than 5 years.

This prospective study performed in Belgium, a low TB incidence country, included 144 children recently exposed to a patient with aTB (smear positive and usually cavitating disease) or with highly suspected aTB, or coming back from a trip to an endemic country. Between 1 and 3 months after their exposure, they were referred to the hospital to investigate whether they were infected with *M. tuberculosis* and for appropriate treatment in case of infection. All children underwent standard clinical assessment with a thorough physical examination, a TST, a chest X-ray and, in case of clinical symptoms suggestive of aTB, the collection of samples from the infection site(s) for microbiological examination. Children were initially classified by the pediatrician as potentially infected or not based on the clinical signs, the initial results of the TST, biological markers of inflammation, the chest X-ray and other radiological exams adapted to symptoms and signs of each child. The follow-up period of the children was at least 3 months after which a second TST was performed in case it was initially negative. Asymptomatic children below 5 years of age with an initial negative TST received a “window prophylaxis” until the second TST was performed. The definitive classification of the children into three groups as (1) non-infected (NI), (2) LTBI, or (3) aTB was only possible when all the results needed for standard classification were available, including the results from the second TST when applicable and those from *M. tuberculosis* culture in case of suspected aTB (Table 1). Fourteen children were considered as non-eligible for the new test evaluation as a consequence of concurrent immunosuppressive treatment (*n* = 2), treatment for TB in the past (*n* = 3) or for more than 5 days for the current episode (*n* = 1), uncertain differential diagnosis between infection with *M. tuberculosis* or with non-typable mycobacteria (*n* = 1), no follow-up for repeated TST (*n* = 3), suspected primary immune deficiency (*n* = 2), HIV infection (*n* = 1), technical drop-out (*n* = 1) (Figure 1).

**Table 1:** Recommended criteria in Belgium for classification of the children [adapted from https://www.fares.be/fr/tbc-infos-pour-professionels/].

	**Symptoms**	**Chest X ray**	**TST (mm induration)**	***M. tuberculosis* culture**	**Response to treatment**
**Uninfected**	Absent	Normal	0–4	–	NR
**LTBI**					
<5 years	Absent	Normal	≥5^*^		NR
≥5 years	Absent	Normal	≥10		NR
**aTB disease**					
<5 years	Present/absent	Abnormal/normal	≥5^*^	±^$^	+
≥5 years	Present	Abnormal/normal	≥5^*^	±	+
	Absent	Abnormal	≥5^*^	±	+

* *In case of recent BCG vaccination after the age of 1 year or in case of repeated BCG vaccination, TST: 5–9 mm is doubtful and TST > 10 mm is clearly positive*.

$ *Confirmed aTB, positive M. tuberculosis culture; suspected aTB, negative M. tuberculosis culture; TST, tuberculin skin test; NR, not relevant*.

Final diagnosis of aTB was based either on microbiological evidence (“confirmed aTB”) or high clinical suspicion (“suspected aTB”). All suspected cases had clinical and/or radiological symptoms and signs consistent with aTB in conjunction with a favorable response to anti-TB treatment. LTBI was defined by a positive TST in an exposed child, without clinical or radiological sign of active disease. The TST was performed using 2 IU of PPD RT23 (Staten Serum Institute, Copenhagen, Denmark). The positivity was defined according to Belgian guidelines^1^, which are in line with those recommended in most European countries (7), i.e., for children in close contact with an aTB index case, 5 mm induration is considered positive in children younger than 5 years and/or with clinical signs of disease. Moreover, the Belgian guidelines recommend to raise the cut-off to 10 mm for children older than 5 years or in case of recent BCG vaccination after the age of 1 year, or in case of repeated BCG vaccination. NI children were all asymptomatic subjects with a TST that remained negative up to 8–12 weeks after the last contact with the aTB index case (Table 1).

### Flow-Cytometric Assay for Specific Cell-Mediated Immune Responses in Activated Whole Blood (FASCIA)

Heparinized blood (250 μl) was collected from all eligible children most often the same day as tuberculin injection, and was processed within 4 h. The FASCIA was adapted from the originally described procedure (19). Briefly, PPD (Statens Serum Institute, Copenhagen, Denmark; 4 μg/ml), ESAT-6 (Lionex, Diagnostics and Therapeutics GmbH, Braunschweig, Germany; 10 μg/ml), CFP-10 (Lionex, Diagnostics and Therapeutics GmbH, Braunschweig, Germany; 5 μg/ml) or HBHA, purified from *Mycobacterium bovis* BCG as detailed elsewhere (19) at 10 μg/ml was added to 400 μl of whole blood diluted 1/10 in enriched RPMI medium (20). Staphylococcal enterotoxin B (SEB, Sigma; 0.5 μg/ml) was added as a positive control and diluted whole blood without antigen represented the negative control. Stimulated and control blood samples were incubated in duplicates (200 μl/well) in 96-well plates for 7 days at 37°C under 5% CO_2_. Cell pellets from duplicates were pooled and then transferred to FACS tubes (BD Biosciences, Erembodegem, Belgium), labeled with anti-CD3-BV421, anti-CD4-APC-H7 and anti-CD8-APC monoclonal antibodies (all from BD Biosciences), and incubated for 15 min at room temperature in the dark. Erythrocytes were lysed (IOTest lysing solution, Beckman Coulter, Analis Belgium) and the pellets were suspended in 500 μl FACSFlow solution (BD Biosciences) before acquisition on a FACSCanto II flow cytometer (BD Biosciences) calibrated daily, and analysis of the data with the Flowjo software (Tree Star, Ashland, OR, USA). The median numbers of CD3^+^ T lymphocytes acquired were 55,000 for PPD, 36,000 for HBHA, 31,000 for ESAT-6 and 36,000 for CFP-10 (25^th^-75^th^ percentiles: 24,000-141,000 for PPD; 18,000-59,000 for HBHA, 19,000-50,000 for ESAT-6 and 21,000-56,000 for CFP-10). The results are expressed as percentages of lymphoblasts among CD3^+^, CD4^+^, or CD8^+^ T cells after deduction of the percentages obtained in the non-stimulated condition. A representative gating strategy is illustrated on the Supplementary Figure.

### Innate Cell Subset Analysis

When the blood volume was sufficient (for 25 children—Supplementary Table), 100 μl of additional undiluted blood was stained *ex-vivo* with different monoclonal antibodies in order to identify monocyte and DC subsets as described (23). Briefly, fresh whole blood was stained for 30 min at room temperature in the dark with monoclonal antibodies to the following surface markers: CD3-FITC, CD19-FITC, CD56-FITC, CD141-PE, CD123-PerCpCy5.5, HLA-DR-PE-Cy7, CD16-APC, CD45-APC-H7, CD11c-V450, and CD14-V500 (all from BD Biosciences, except anti-CD141 from Miltenyi and anti-CD45 from Biolegend). After lysis of the red blood cells with 2 ml of BD-lysing solution (BD Biosciences), data were acquired on a FACSCanto II and analyzed with the Flowjo software. The median number of acquired CD45^+^ cells was 172,000 (25th−75th percentiles = 144,000–208,000). A sequential gating strategy was applied as described (23), allowing us to identify the three subsets of monocytes (CD14^+^CD16^−^, CD14^+^CD16^+^, CD14^−^CD16^+^) and of DCs (CD123^+^CD11c^−^ plasmacytoid DC (pDC), CD123^−^CD11c^+^CD141^−^ type 1 myeloid DC (mDC) or CD123^−^CD11C^+^CD141^+^ type 2 mDC). The results of the different monocyte subsets and of the pDC and mDC subsets are expressed as percentages among CD45^+^ cells. The results of type 1 and type 2 mDC are expressed as percentages among total mDC (CD123^−^CD11c^+^).

### Statistical Analyses

GraphPad Prism version 5 for Windows (GraphPad Software,San Diego, CA, USA, www.graphpad.com) was used for statistical analyses. Comparisons of continuous variables between groups were performed with the non-parametric Kruskal-Wallis test combined with the Dunn's multiple comparison. A Mann-Whitney test was used when comparisons were limited to two groups of results. A *p*-value < 0.05 was considered significant. Receiver Operating Characteristic (ROC) curves were established to determine optimal cut-off values.

## Results

### Characteristics of the Study Population

As detailed above, 130 children were eligible for the evaluation of the index tests, and when all the results were available, including a second TST in case of negativity of the first one, and the microbiological results, they were classified according to reference standard as aTB (*n* = 33 or 25% of included children), LTBI (*n* = 28, 22%) or *M. tuberculosis* exposed but NI children (*n* = 69, 53%). Children with aTB were further classified in two subgroups: localized aTB (*n* = 23, 70% of the aTB cases; pulmonary TB except one case of TB lymphadenitis) and severe/disseminated aTB (*n* = 10, 30% of the aTB cases; miliary TB, central nervous system aTB including meningitis, severe pulmonary TB associated with abdominal and/or cervical lymphadenitis, hence with multiple sites involved). Among children with aTB, 16 of them had confirmed aTB whereas the other 17 were classified as suspected aTB. Table 2 shows the demographic and clinical characteristics of the children. Noteworthy, 20/33 children (60%) with aTB were younger than 3 years of age, the median age and inter-quartiles (IQR) of children with aTB being 24 months (8–72 months), whereas children with LTBI were older (median age: 96 months; IQR: 81–135 months). This difference between the two groups of infected children was significant (*p*: 0.0018) and consistent with the age-dependent risk of developing active disease. Of note also is the fact that the large majority of children in all groups were not BCG vaccinated. BCG vaccination is not recommended in Belgium and the vaccine is not even available anymore. The only exception to this recommendation is for children below 5 years of age with a migration background from a high TB endemic country. In this case, it is recommended to get the vaccine from a neighboring country (France).

**Table 2:** Demographic and clinical data from included children.

	**Uninfected**	**LTBI**	**aTB**
	***n* = 69**	***n* = 28**	***n* = 33**
Age in months: median (IQR)	10 (6–36)	96 (81–135)	24 (8–72)
**Gender: % F**	44	43	27
**Ethnic origin**, ***n*****°**			
Belgium	9	7	8
Northern Africa (Maghreb)	17	10	12
Middle East	1	0	1
Central/Western/Eastern Africa	14	6	9
Eastern Europe	7	5	2
Asia	2	0	1
Other/unknown	19	0	0
**Country of birth**			
Belgium	42	20	30
Spain	0	1	0
Northern Africa (Maghreb)	3	1	2
Central/Eastern/Western Africa	4	2	0
Eastern Europe	2	4	0
Asia	0	0	1
Other/unknown	18	0	0
**BCG vaccinated:** ***n*****° (%)**	9 (13)	5 (18)	1 (3)
**Nature of exposure:**			
Parent *n*° (%)	19 (27)	6 (21)	10 (30)
Family member other than parent/close friend *n*° (household n°)	0	10 (3)	8 (2)
School/Preschool *n*°	1	5	3
Trip to endemic country/social situation *n*°	14	3	12
Recent arrival from endemic country *n*°	3	4	0
Non-household contact	27	0	0
Adult index patient initially treated for suspected TB (non-confirmed)	5	0	0
**Initial TST result (diagnostic evaluation and treatment initiation):** ***n*****° positive (%)**	0	27 (96)	30 (90)
**Definitive TST result (3 months after last contact):** ***n*****° positive (%)**	0	28 (100)	33 (100)
Median (mm induration)	0	16	14
IQR	0	13–22	10–20
**Chest-X-ray abnormalities** ***n*****° (%)**	NA	0	32 (96)
Gangliopulmonary disease			25
Miliary pattern			5
Cavernous disease (adult form)			1
Pleural effusion			1
**Microbiology**			
Smear + PCR + Culture +			1
Smear – PCR + Culture -			3
Smear-PCR + Culture +			2
Smear – PCR – Culture +			10
Total confirmed by microbiology *n*° (%)			16 (48)
**Sample of microbiological confirmation**			
Gastric aspirate			10
Biopsy			5
Broncho-alveolar lavage			1
**Localized TB—total** ***n*****°:**			23
Pulmonary lymph node TB *n*° (culture confirmed: *n*°- %)			22 (6–27)
TB lymphadenitis (culture confirmed)			1
Disseminated TB -all culture confirmed but 1 ^*^ total ***n*****°:**			10
Miliary (pulmonary) TB			3
Miliary TB with associated cerebral tuberculomas			2
TB meningitis			1
Both extensive abdominal and cervical TB lymphadenopathy, associated with pulmonary lesions^*^			3^*^
Pulmonary TB + extrapulmonary (cervical) TB lymphadenopathy			1

* *, culture negative*.

### Identification of *M. tuberculosis*-Infected Children by Antigen-Specific CD3^+^ FASCIA, Active, and Latent Stages of Disease Confounded

**Figure 2:**
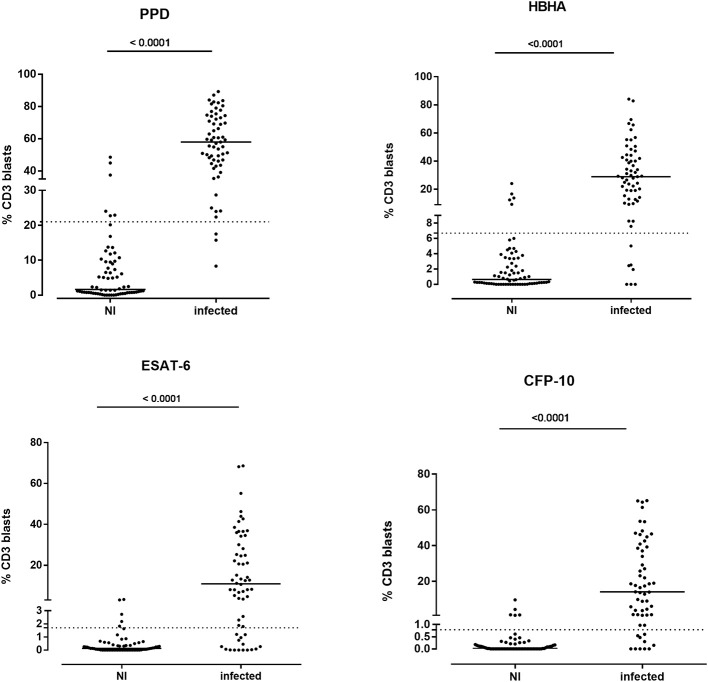
Percentages of CD3^+^ blasts induced by mycobacterial antigens in *M. tuberculosis*-infected and in non-infected children. Ten-fold diluted whole blood was incubated during 7 days at 37°C with PPD (4 μg/ml), HBHA (10 μg/ml), ESAT-6 (10 μg/ml), or CFP-10 (5 μg/ml), as indicated, before labeling the cells with anti-CD3 monoclonal antibody and analysis of the percentages of CD3^+^ blasts by flow cytometry. Each symbol represents the value from an individual subject after deduction of the percentage obtained in non-stimulated condition, and the horizontal lines indicate the medians. The dotted horizontal lines indicate the cut-off values determined by ROC curve analysis. Values obtained for non-infected (NI) children were compared to those from infected children (LTBI/aTB) by Mann-Whitney *U* test.

As the identification of *M. tuberculosis*-infected children (either with LTBI or with aTB) is the first recommended step in the systematic evaluation of children exposed to a case of aTB (1, 7), we first compared the results of the mycobacterial antigen-induced blast cells obtained for all infected children (LTBI + aTB) to those of the NI children. The percentages of antigen-specific CD3^+^ responses analyzed by FASCIA provided a strong discrimination between the two groups for all four antigens, PPD, HBHA, ESAT-6, CFP-10, used *in vitro* to expand specific blast cells (*p* < 0.0001) (Figure 2). Cut-off values of the percentages of CD3^+^ blasts were determined for each antigen by ROC curves established by comparing results from *M. tuberculosis*-infected children with those from NI children. The area under-operating-characteristic curves (AUC) were comprised between 0.88 and 0.98, and we set the thresholds to each antigen to achieve a balanced sensitivity and specificity (Table 3).

**Table 3:** Diagnostic accuracy of the FASCIA.

	**AUC**	**Cut-off %**	**Sensitivity (95% CI)**	**Specificity (95% CI)**	**Likelihood ratio**
**Infected (LTBI/aTB) vs. non-infected**					
PPD-CD3^+^ blasts	0.98	21.3	95.1% (82.3–99.0)	91.2% (81.8–96.7)	10.8
HBHA-CD3^+^ blasts	0.90	6.7	88.5% (77.8–95.3)	92.9% (84.1–97.7)	10.3
ESAT-6-CD3^+^ blasts	0.88	1.7	75.0% (62.1–85.3)	92.8% (83.9–97.6)	10.3
CFP-10-CD3^+^ blasts	0.90	0.79	82.5% (70.1–91.3)	92.8% (83.9–97.6)	11.1
**aTB vs. LTBI**					
CFP-10-CD3^+^ blasts	0.71	0.79	93.1% (77.2–99.1)	32.1% (15.8–52.3)	1.3
CFP-10-CD4^+^ blasts	0.67	1.05	93.1% (77.2-99.1)	39.2% (21.5-59.4)	1.5
PPD-CD8^+^ blasts	0.76	10.94	42.4% (25.4–60.7)	96.4% (81.6–99.9)	11.8
CFP-10-CD8^+^ blasts	0.77	8.29	41.3% (23.5–61.0)	96.4% (81.6–99.9)	11.6

*AUC, area under the curve*.

**Figure 3:**
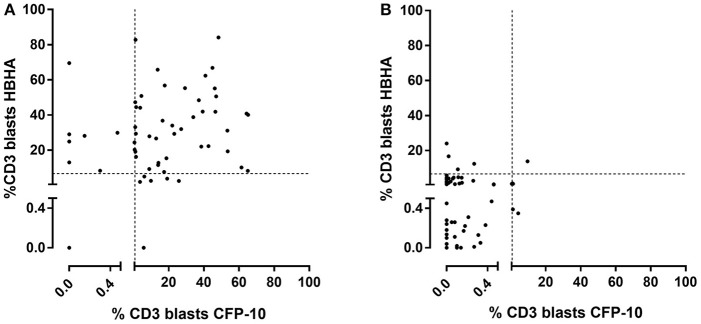
Combined analysis of the percentages of HBHA- and CFP-10-induced CD3^+^ blasts in *M. tuberculosis*-infected children (LTBI/aTB). Ten-fold diluted whole blood was incubated during 7 days at 37°C with HBHA (10 μg/ml) or CFP-10 (5 μg/ml), as indicated, before labeling the cells with anti-CD3 monoclonal antibody and analysis of the percentages of CD3^+^ blasts by flow cytometry. **(A)** Results from *M. tuberculosis-*infected children (LTBI and aTB combined), and **(B)** results from non-infected children, each symbol representing the value obtained for an individual subject after deduction of the percentage obtained in the non-stimulated condition. The dotted lines indicate the cut-offs chosen for the two tests.

However, in spite of the highly significant differences between the group of infected children as a whole (LTBI/aTB) compared to the group of NI children, the antigen-specific CD3^+^ FASCIA did not allow us to identify all the LTBI/aTB children when we analyzed only separately the responses induced by one of the four mycobacterial antigens tested. The results obtained in response to ESAT-6 were the worse and were therefore not further considered. In contrast, the PPD-induced CD3^+^ blasts provided the highest sensitivity (95.1%) for the detection of *M. tuberculosis-*infected children (LTBI/aTB), as only 3/61 of them were not detected at the selected cut-off for positivity (2 LTBI and 1 aTB). The specificity was 91.2% in this specific cohort of children, but this test would probably be of more limited value in cohorts of BCG-vaccinated children (Table 3). Interestingly, combining the percentages of HBHA-induced CD3^+^ blasts with those of CFP-10-induced CD3^+^ blasts resulted in the detection of all but one LTBI/aTB children, providing thus 98.2% sensitivity with 86.9% specificity for the detection of *M. tuberculosis*-infected children among *M. tuberculosis*-exposed children (Figure 3). Whereas, 9/69 NI children were misclassified by combining the results of these two tests (specificity of 86.9%), both tests were positive for 41/57 *M. tuberculosis*-infected children (LTBI/aTB), and one or the other test was positive for an additional 15 *M. tuberculosis-*infected children, leading to the identification of 56/57 LTBI/aTB children (sensitivity of 98.2%) (Figure 1). The only child who remained undetected by this combination was a 1-month-old child with suspected perinatal infection and with a negative TST at inclusion, which became positive 3 months later. These tests thus allowed us to detect at inclusion 3 children who in spite of clinical signs suggestive of aTB had a negative TST at initial diagnosis and treatment initiation, comforting the clinician in his clinical suspicion: a 3-months old child with culture-confirmed miliary TB, a 1.5-year-old child with culture-confirmed TB meningitis, and a 6-years old immune-competent boy with pulmonary TB. This combination of tests appears thus useful to detect both young and older *M. tuberculosis* infected children as confirmed by a separate analysis of the results for children younger or older than 5 years of age (data not shown).

Measuring the percentages of PPD-, HBHA-, ESAT-6-, or CFP-10-induced blasts among the CD4^+^ or the CD8^+^ cells provided no added value for the identification of LTBI/aTB children (data not shown).

### Added Value of Antigen-Specific CD8^+^ FASCIA for the Discrimination Between Children With LTBI and Those With aTB

**Figure 4:**
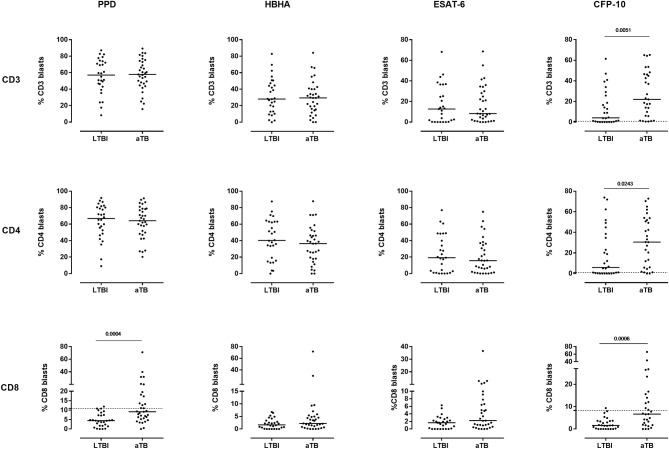
Comparison of the percentages of T cell blasts induced by mycobacterial antigens between children with LTBI and those with aTB. Ten-fold diluted whole blood was incubated during 7 days at 37°C with PPD (4 μg/ml), HBHA (10 μg/ml), ESAT-6 (10 μg/ml) or CFP-10 (5 μg/ml), as indicated, and the cells were labeled with anti-CD3, anti-CD4, and anti-CD8 monoclonal antibodies. The percentages of CD3^+^ (upper panels), CD4^+^ (middle panels) and CD8^+^ (lower panels) blasts were determined by flow cytometry. Each symbol represents the value from an individual child after deduction of the percentage obtained in the non-stimulated condition. The horizontal filled lines indicate the medians. For the tests providing statistically significant differences between the two groups of infected children, horizontal dotted lines were added to indicate the cut-off of positivity. Statistical analysis was performed by Mann-Whitney *U* test. LTBI, latently TB infected; aTB, active tuberculosis.

Among the percentages of CD3^+^ and CD4^+^ blasts, only those induced by CFP-10 resulted in higher median values in children with aTB compared to those with LTBI (*p* = 0.0051 and *p* = 0.0243 for CD3^+^ and CD4^+^ blasts, respectively) (Figure 4, upper and middle right panels). However, high overlaps were noticed between the results of these two groups of children, resulting in a poor diagnostic accuracy of these tests to differentiate children with LTBI from those with aTB (Table 3).

In contrast, the analysis of the percentages of PPD- and CFP-10-induced CD8^+^ blasts was of further interest. They were both significantly higher in children with aTB compared to those with LTBI (*p* = 0.0004 and *p* = 0.0006 for PPD and CFP-10, respectively). In these cases high percentages of blasts cells were found only in children with aTB (Figure 4, lower panels). To answer the clinical question, i.e., to confirm the correct classification as LTBI of asymptomatic children with normal chest X ray, and as aTB in children with clinical symptoms and/or radiological signs of disease, thresholds of positivity were selected by ROC curve analysis to achieve optimal specificity (Table 3). Results obtained for the PPD- and the CFP-10-CD8^+^ FASCIA were very similar, albeit slightly better for PPD (Table 3), and combining the results from the two tests did not improve their diagnostic accuracy (data not shown).

**Figure 5:**
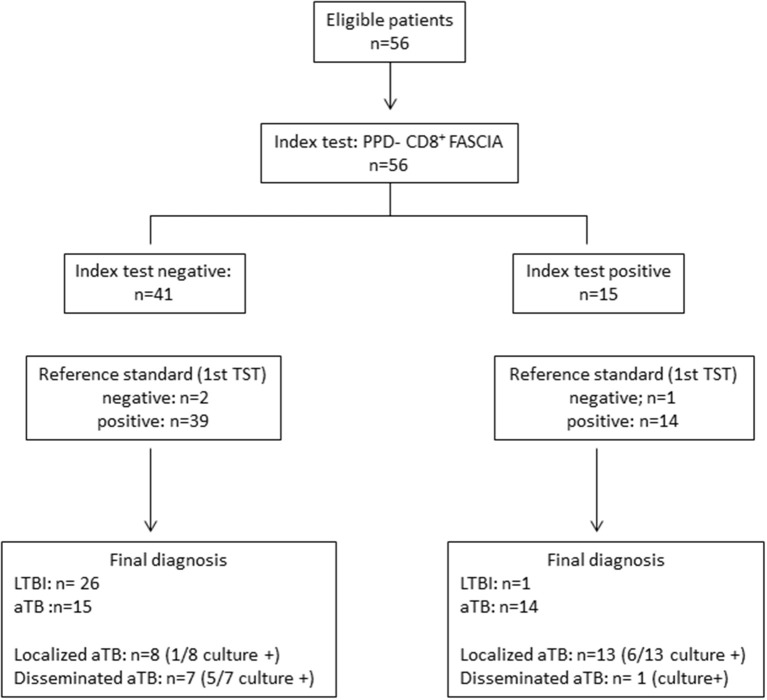
STARD diagram reporting the flow of *M. tuberculosis-*infected children (LTBI/aTB) for the evaluation of the PPD-induced CD8^+^ blasts lymphocytes for the identification of children with aTB on the one hand and those with LTBI on the other hand. The reference standard test was the tuberculin skin test (TST) performed in a first diagnostic stage at the time of inclusion in the study. This diagram reports results obtained for the 56 *M. tuberculosis*-infected children (LTBI/aTB) detected by the combined HBHA- and CFP-10-induced CD3^+^ FASCIA as reported in Figure 1.

The results of the PPD-CD8^+^ FASCIA with values above the threshold allowed us to correctly identify 13/21 (61.9%) of the children with localized aTB, with a specificity for localized aTB of 96% as only 1/27 LTBI was misclassified (Figure 5). These results may therefore reassure the clinician in the decision to start a tri- or quadritherapy based on his/her clinical evaluation, especially in cases of initial negative TST (one child in this cohort). The results of the PPD-CD8^+^ FASCIA were, in contrast, of little help for the identification of children with disseminated aTB, as only 1/8 showed a value above the defined threshold (Figure 5). Children with disseminated aTB had indeed lower percentages of PPD-induced CD8^+^ blasts compared to those with localized aTB (*p* = 0.0111). Of note is that the positivity of the PPD-CD8^+^ FASCIA was not associated to the positivity of the *M. tuberculosis* culture (Figure 5).

For the asymptomatic children classified as LTBI by the clinician, the values of the PPD-CD8^+^ FASCIA below the threshold allowed us to correctly identify 26/27 LTBI children, whereas only one child classified as LTBI based on clinical, radiological and TST results, had a PPD-CD8^+^ FASCIA value slightly above the threshold and was therefore misclassified (Figure 5). However, the specificity for the identification of LTBI was poor (63.5%) as 15/41 children with a negative test had in fact aTB, but these children were most often identified by their clinical symptoms.

### Potential Added Value of Blood DC Subsets for the Identification of Infected Children With LTBI

**Figure 6:**
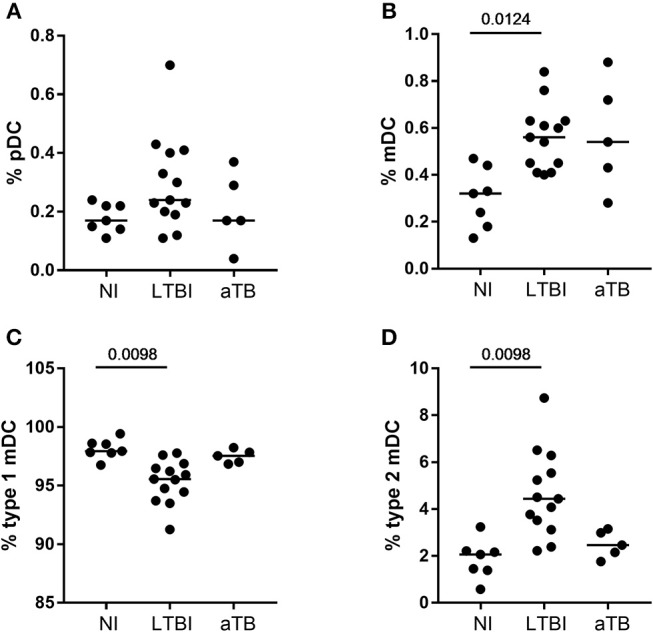
Proportions of circulating plasmacytoïd and myeloïd DC subsets in *M. tuberculosis*-infected compared to non-infected children. **(A,B)** The proportions of pDC and of total mDC were evaluated in the peripheral blood of non-infected children (NI), children with a latent TB infection (LTBI), or with active TB (aTB) and the results were expressed as percentages among CD45^+^ cells (upper panels). **(C,D)** The proportions of type 1 and type 2 mDC subsets are those among the total mDC (lower panels). Horizontal lines represent the medians of percentages. Statistical analysis was performed by a Kruskal-Wallis test followed by Dunn's tests.

As the proportions of blood monocytes and DC subsets were reported to be altered during *M. tuberculosis* infection in adults (23), we conducted a pilot study for 25/130 children to characterize these parameters, as this can be done by flow cytometry on a small blood volume. In contrast to adults, the proportions of the three different subsets of monocytes identified by their CD14 and CD16 expression were not different between NI children and infected children (LTBI or aTB) (Supplementary Table). The proportions of blood pDC among CD45^+^ cells were also similar between the 3 groups of children, whereas those of mDC were higher among children with LTBI compared to NI children (*p* = 0.0124, Figure 6, upper panels). Among mDC, the proportions of type 2 mDC were significantly higher and those of type 1 mDC were lower in children with LTBI compared to NI children (*p* = 0.001, Figure 6, lower panels). Moreover, when we focused on infected children and compared the percentages of type 2 mDC between children with a final diagnosis of LTBI or aTB, they were significantly higher among children with LTBI (*p* = 0.0098, Mann-Whitney *U* test). These results suggest that high percentages of blood type 2 mDC is a feature of children with LTBI as 10/13 children with a final diagnosis of LTBI were identified at a 3.25 % cut-off.

## Discussion

Whereas effective management of children in contact with an aTB index case, essentially household contacts, was recently recommended, as it could substantially reduce childhood disease and death caused by TB (6), effective application of this recommendation remains a challenge, partially due to the lack of efficient diagnostic tests adapted to children and especially to infants. The latter are at high risk to rapidly develop severe and disseminated aTB when infected (3) and a rapid diagnosis is difficult, as it is essentially based on clinical symptoms, clinical and radiological signs and TST results. Diagnosis of *M. tuberculosis* infection remains a relevant part of the diagnosis of both aTB and LTBI. Whereas in >5 years old children commercial IGRA are recommended even for the diagnosis of aTB (24), the sensitivity of both IGRA and TST is reduced in <2 years old children, and the IGRA results are often not interpretable before 1 year of age and in cases of central nervous system disease (24).

We report here the identification of *M. tuberculosis-*infected children (LTBI/aTB) with a test based on the appearance of lymphoblasts in whole blood stimulated with mycobacterial antigens. These lymphoblasts result from lymphocyte activation by mycobacterial antigens, and do not appear in *M. tuberculosis*-exposed but non-infected children. In contrast, combining the lymphoblast appearance among CD3^+^ lymphocytes in response to two different mycobacterial antigens, such as CFP-10 and HBHA, allowed us to detect 56/57 of the infected children among the 126 children evaluated with these two tests. The 98.2% sensitivity of this combined test associated with 86.9% specificity approaches the WHO recommendations for the development of new diagnostic tests for TB (25). Interestingly, if 9 NI children were misclassified resulting in a 86.9% specificity, 8 of them had been in very close and prolonged contact with a parent (7/8, usually the mother) or grandparent (1/8) with pulmonary aTB, and the ninth one was a newborn from a mother treated for pulmonary aTB during the last trimester of pregnancy. All 9 were younger than 5 years old at their diagnostic evaluation and therefore received prophylaxis during the “window of uncertainty,” that is up to 3 months after the last contact with index case, according to the Belgian guidelines. None of them developed signs of disease, nor positive TST at follow-up, up to the final evaluation that took place at least 3 months after the initial evaluation, but up to the age of 6 months for the youngest infants. They were thus all nine classified as NI but the prophylactic treatment probably played a role in their good clinical evolution, and may have skewed the natural course of the *M. tuberculosis* infection. The calculated specificity of this new combined test is therefore probably underestimated.

One major advantage of this test is that it only requires 250 μl whole blood and that its readout, the lymphoblast appearance, is IL-2 dependent in contrast to IGRA that depend on IFN-γ secretion, and require substantially more blood. As neonatal CD4^+^ lymphocytes synthesize appreciable amounts of IL-2 (15), whereas they are variably impaired in their IFN-γ production (16), this new test may be ideal to diagnose *M. tuberculosis* infection during infancy. It could also be of value to diagnose congenital or neonatal TB that result in overwhelming sepsis and death when the diagnosis is delayed (26), and that occurs as a consequence of aTB in pregnant women, which is frequent in high burden countries.

Other immunological approaches had already been evaluated to identify *M. tuberculosis*-infected children as a whole (both aTB disease and LTBI) or to more precisely identify children with aTB. Most of them are based on the measurement of one or different cytokines released after *in vitro* whole blood stimulation with mycobacterial antigens, either PPD or ESAT-6 and CFP-10, the antigens present in QuantiFERON tubes. Some studies highlighted the diagnostic potential of ESAT-6- and CFP-10-induced IP-10, but studies performed in children reported no or very little added value of IP-10 over IFN-γ except that IP-10 is released independently of age (18, 26–28). Only one study, performed in adults, reported superiority of IP-10 over IFN-γ induced by ESAT-6 and CFP-10, probably because IP-10 concentrations were measured after 3 days of *in vitro* stimulation with the antigens instead of the classical 24 h stimulation. In these conditions, IP-10 concentrations provided 100% sensitivity for the diagnosis of aTB, but the sensitivity for LTBI was only 70% (29). Some of these studies also pointed out the potential interest of measuring IL-2 concentrations, with PPD-induced IL-2 concentrations providing even a more accurate discrimination between infected and uninfected children than mycobacterial antigen-induced IP-10 in the small cohort of children investigated by Tebruegge et al. (18), with 100% sensitivity and 96% specificity (18). Our results are in line with this study, as the antigen-induced blast cell development is essentially IL-2 dependent. However, we extended the interest of mycobacterial antigen-induced IL-2-mediated immune function to a larger cohort of children comprising among others 20 children with aTB who were younger than 3 years.

If the identification of *M. tuberculosis-*infected children (LTBI/aTB) remains an important step of *M. tuberculosis* contact management in childhood, differential diagnosis between aTB and LTBI is a major second step to guide treatment strategy. However, this distinction remains often difficult to establish, and the classical immunological tests, TST or IGRA do not provide any help for this distinction. A pilot study performed on a very small number of infected children (6 LTBI et 8 aTB) suggested a possible discrimination between LTBI and aTB by the ratio between the concentrations of TNF-α and IL-2 induced by ESAT-6 and CFP-10, a low ratio being in favor of LTBI (88% sensitivity and 83% specificity) (30). In contrast, a high number of IL-2-secreting cells detected by ELISPOT after *in vitro* stimulation with another mycobacterial antigen, Rv2780, was suggested to facilitate the identification of children with aTB, at least among >5 years old children (17). On the other hand, the results reported by Tebruegge et al. (18) indicated that only a combination of cytokines may help to differentiate aTB from LTBI in children and the best combination they found within a small cohort of children older than 10 years was the measurement of both TNF-α and IL-10 concentrations induced by PPD. The main limitation of these studies is that measurement of cytokine concentrations is usually performed by batches on several samples and therefore not really suitable for individual diagnostic purposes.

To avoid this, other studies were based on readouts analyzed by flow cytometry that are more convenient for individual testing but that most often are expensive and need highly trained personnel. This was the case for a T cell activation marker assay based on a ratio between the mean fluorescence intensity (MFI) of the labeling of the CD27 surface marker of *M. tuberculosis*-specific CD4^+^ cells containing IFN-γ compared to the MFI of this marker expressed by all the CD4^+^ T lymphocytes of the blood sample. This ratio was higher for children with aTB than in non-aTB children and identified children with aTB with 83.3 % sensitivity and 96.8% specificity among a cohort of children with suggestive symptoms living in a high TB incidence country (31). Another flow cytometry based study reported the identification of young children with LTBI (<3 years) by their high proportion of CD4^+^ T lymphocytes producing only IL-17 in response to HBHA, and the identification of older children with LTBI by their high ratio of CD4^+^ lymphocytes producing only IFN-γ over those producing only TNF-α in response to ESAT-6 (32).

In this study, we took advantage of flow cytometry, allowing us to work on an individual basis and with small blood volumes, and we used as a first readout the analysis of the blast lymphocyte appearance induced by mycobacterial antigens and detected by the analysis of the size and granularity of these cells. Labeling with monoclonal antibodies was limited to a classical phenotype (CD3—CD4—CD8) routinely performed in most clinical laboratories. The combination of HBHA- and of CFP-10-induced blasts provided an excellent discrimination between infected (LTBI/aTB) and NI children. The distinction between children with LTBI and those with aTB was best achieved by analyzing the PPD-induced CD8^+^ blasts, but was less performant. Nevertheless, this test allowed us to identify 61.9% (13/21) of the children with localized aTB, the *M. tuberculosis* culture being positive in only 46% of them. The diagnostic accuracy of this test was lower for disseminated compared to localized aTB, perhaps as a consequence of high immune suppression in disseminated aTB. A specific induction of CD8^+^ T cell responses by mycobacterial antigens during aTB is in line with previous reports (33, 34). The mechanisms of *in vivo* induction of these cells are still incompletely understood (33) This immunological signature was reported to be generated in response to a high bacillary load (35, 36), which was not always the case in our study, as less than half of children with aTB were culture confirmed.

In contrast to children with aTB, those with LTBI had low percentages of PPD-induced CD8^+^ blasts, so that a high proportion of such cells in an asymptomatic child should increase the index of suspicion for possible aTB and prompt the clinician to re-evaluate the child for signs of active disease. Our pilot study on the characterization of blood DC subsets further identified a low proportion of type 2 mDC as another potential biomarker of LTBI in children. These results suggest that the analysis of the percentages of blood type 2 mDC could help to identify children with LTBI among children exposed to an aTB index case. However, these altered proportions of blood DC subsets differ from those previously reported in adults (23, 37), likely reflecting a difference in the pathogenesis of *M. tuberculosis* infection in children and in adults. Further studies should be performed to confirm this message and to evaluate the diagnostic accuracy of the combined determination of the percentage of PPD-induced CD8^+^ blasts and of the type 2 mDC for the optimal differential diagnosis between aTB and LTBI in children. Of note is that the only child with LTBI identified in this study with a high percentage of PPD-induced CD8^+^ blast was characterized by a high proportion of type 2 mDC, suggesting the added value of combining these two tests.

The limitations of this study are mostly due to the fact that it was performed in a low TB incidence country with a very low BCG vaccination coverage. As a consequence, the size of the different sub-groups was relatively small, and the diagnostic value of the blast cell responses to PPD might be overestimated if the test were performed on BCG-vaccinated children. Moreover, the DC subset analysis was a pilot study. Our results should therefore be confirmed on larger cohorts of children, preferably in a high TB incidence country with high BCG vaccination coverage. Other limitations are the necessary long-term *in vitro* stimulation with antigens (7 days) for the FASCIA test, delaying the conclusions for 1 week after blood sampling, and the current absence of commercially available HBHA. Additional complementary approaches could be the measurement of chemokines, especially IP-10, on antigen-stimulated whole blood culture supernatants that may be collected just before the analysis of the blast cells on the pellets (29), and/or the analysis of CD27 expression by blast cells. Finally, the absence of head-to-head comparison of the results of the FASCIA to those obtained with a commercial IGRA may be perceived by some as a major limitation of this study, as it did not allow us to demonstrate a better accuracy of the FASCIA compared to the IGRA. We have chosen here to compare the diagnostic accuracy of the FASCIA to that of the TST, which is still the reference test for detection of *M*. *tuberculosis* infection in a low TB incidence country with extremely low BCG-vaccination coverage as is the case in Belgium. Indeed, TST has excellent specificity in non-vaccinated children (98%), and significantly higher sensitivity than commercial IGRA for the diagnosis of LTBI (82 vs. 73%) (11).

To our knowledge, this study is the first to provide a potential diagnostic test with 98 % sensitivity for the detection of *M. tuberculosis*-infected children (LTBI/aTB), even in very young infants, on a blood volume as small as 250 μl, compared to 93.4 % sensitivity of the TST in the same cohort. In the absence of a perfect test to diagnose *M. tuberculosis* infection in young children, the FASCIA may play a valuable part in concert with TST and possibly IGRA for the initial steps in diagnosis of most *M. tuberculosis-*infected children among those exposed to an aTB index case. The additional partial discrimination between aTB and LTBI provided by the FASCIA may provide further arguments for the clinicians to help them in the classification of the children in the two different subgroups of infection. However, further studies should be performed to evaluate the performance of the FASCIA to discriminate *M. tuberculosis* infection from other diseases.

## Ethics Statement

The protocol for this study received approval from the ethics committee ULB-Hôpital Erasme and informed written consent was obtained from all parents.

## Author Contributions

AD, SD, ID, M-KF, AM, and FMo: patient's inclusion. AD, VD, KS, VC, and FMa: conception and design. AD, VD, VC, and AV: acquisition and analysis of data. MS and CL purification of the mycobacterial antigens. AD, VD, and FMa: drafting of the manuscript. VD, VC, AM, FMo, CL, and FMa: revising the manuscript for important intellectual content.

### Conflict of Interest Statement

The authors declare that the research was conducted in the absence of any commercial or financial relationships that could be construed as a potential conflict of interest. The reviewer RBR declared a past co-authorship with one of the authors MS to the handling editor.

## Footnotes

^1^https://www.fares.be/fr/tbc-infos-pour-professionels/
