# Supplementary material for: Identification of Mycobacterium tuberculosis Infection in Infants and Children With Partial Discrimination Between Active Disease and Asymptomatic Infection
Source: Front Pediatr. 2019 Jul 25;7:311. doi: 10.3389/fped.2019.00311 (PMC6669376; doi:10.3389/fped.2019.00311)
Supplement: Supplementary file 1 [file Table_1.DOCX]

Supplementary material

Table: Children included in the phenotypic analysis of monocytes and dendritic cells subsets

NI LTBI aTB

Total number 7 13 5

Median age (months) 24 96 84

Age (months) IQR 18-42 84-108 24-90

Disseminated aTB disease (% of aTB) - - 20

Monocyte subsets (median % and IQR)

CD14^+^CD16^-^ 8.5 (3.6-8.8) 8.0 (7.6-10.5) 6.7 (6.7-8.9)

CD14^+^CD16^+^ 0.3 (0.2-0.5) 0.3 (0.2-0.5) 0.3 (0.3-0.4)

CD14^-^CD16^+^ 1 (0.8-1.6) 0.9 (0.8-1.2) 1.5 (1.4-1.7)
