# Supplementary figures and images for: Identification of Mycobacterium tuberculosis Infection in Infants and Children With Partial Discrimination Between Active Disease and Asymptomatic Infection
Source: Front Pediatr. 2019 Jul 25;7:311. doi: 10.3389/fped.2019.00311 (PMC6669376; doi:10.3389/fped.2019.00311)

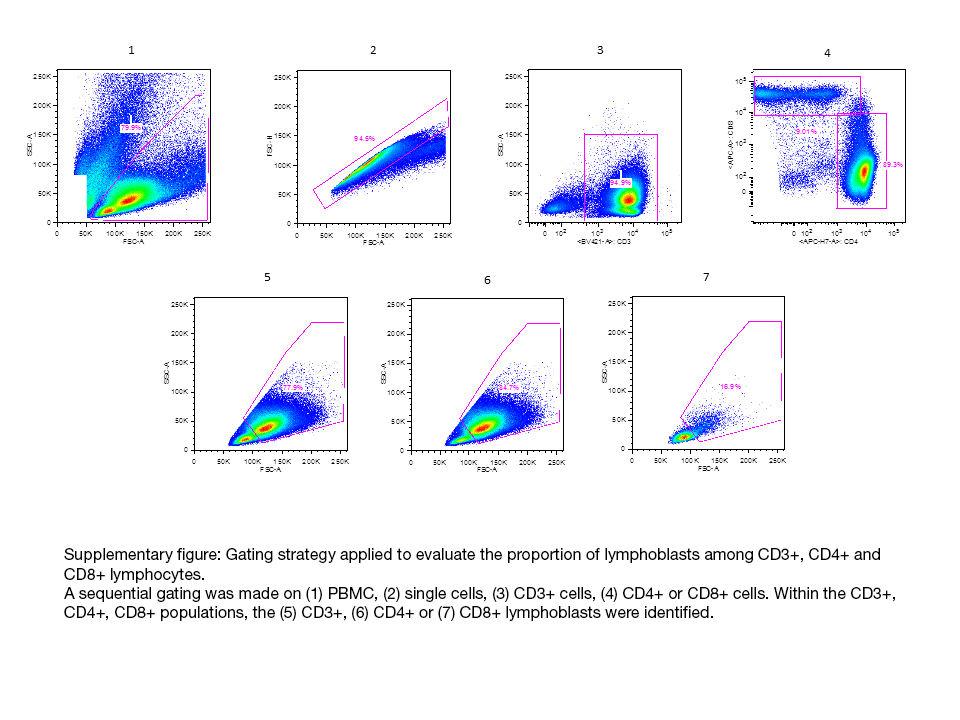

Supplement: Supplementary file 2 [file Image_1.tif]
